# The Role of TL1A and DR3 in Autoimmune and Inflammatory Diseases

**DOI:** 10.1155/2013/258164

**Published:** 2013-12-21

**Authors:** Yoshihiro Aiba, Minoru Nakamura

**Affiliations:** ^1^Clinical Research Center, National Hospital Organization Nagasaki Medical Center, Kubara 2-1001-1, Omura 856-8562, Japan; ^2^Department of Hepatology, Nagasaki University Graduate School of Biomedical Sciences, Kubara 2-1001-1, Omura 856-8562, Japan; ^3^Headquarters of PBC Research in NHOSLJ, Clinical Research Center, National Hospital Organization Nagasaki Medical Center, Kubara 2-1001-1, Omura 856-8562, Japan

## Abstract

TNF-like ligand 1A (TL1A), which binds its cognate receptor DR3 and the decoy receptor DcR3, is an identified member of the TNF superfamily. TL1A exerts pleiotropic effects on cell proliferation, activation, and differentiation of immune cells, including helper T cells and regulatory T cells. TL1A and its two receptors expression is increased in both serum and inflamed tissues in autoimmune diseases such as inflammatory bowel disease (IBD), rheumatoid arthritis (RA), and ankylosing spondylitis (AS). Polymorphisms of the *TNFSF15* gene that encodes TL1A are associated with the pathogenesis of irritable bowel syndrome, leprosy, and autoimmune diseases, including IBD, AS, and primary biliary cirrhosis (PBC). In mice, blocking of TL1A-DR3 interaction by either antagonistic antibodies or deletion of the DR3 gene attenuates the severity of multiple autoimmune diseases, whereas sustained TL1A expression on T cells or dendritic cells induces IL-13-dependent small intestinal inflammation. This suggests that modulation of TL1A-DR3 interaction may be a potential therapeutic target in several autoimmune diseases, including IBD, RA, AS, and PBC.

## 1. Characteristics of TL1A and DR3

### 1.1. TL1A

TL1A, also referred to as vascular endothelial growth inhibitor (VEGI)-251, is a member of the tumor necrosis factor superfamily (TNFSF) of ligands, which was identified by Migone et al. in 2002 [1]. Although TL1A was identified as a longer variant of *TL1/VEGI*, the fourth exon of *TL1A* encodes the majority of *TL1/VEGI*, and it has been presumed that the original TL1/*VEGI* was a cloning artifact. TL1A exhibits approximately 20–30% homology to other TNFSF members [1]. Human TL1A consists of 251 amino acids: 35 in the cytoplasmic domain, 24 in the transmembrane region, and 192 in the extracellular domain. There are two potential N-linked glycosylation sites in the TL1A amino acid sequence, specifically Asn residues at amino acids 133 and 229 [1]. TL1A is a type II transmembrane protein. TL1A is initially expressed as a membrane-bound protein and is subsequently released as a soluble protein via ectodomain shedding by a metalloproteinase such as TNF-*α* converting enzyme (TACE) [2, 3]. TL1A expression is detected on human umbilical vein endothelial cells and synovial fibroblast-like cells and is upregulated by stimulation with proinflammatory cytokines such as TNF-*α*, IL-1, and PMA, a phorbol ester known to be a potent activator of protein kinase C [1, 4]. TL1A expression has also been confirmed on antigen-presenting cells and lymphocytes that are activated by Toll-like receptor (TLR) ligands, enteric bacteria, and Fc*γ* receptor (Fc*γ*R) crosslinking [5–7].

### 1.2. DR3

DR3, also known as APO-3, TRAMP, LARD, and WSL-1, is a member of the tumor necrosis factor receptor superfamily (TNFRSF) with a typical death domain that consists of an approximately 60-amino-acid globular bundle of 6 conserved *α* helices found in the cytoplasmic region. Although DR3 is most homologous to TNFR1, which is widely expressed, its expression is mostly restricted to lymphocytes such as NK cells and T cells, in particular NKT cells and is enhanced upon their activation [8–10]. DR3 is more highly expressed on Th17 cells than on Th1 and Th2 cells, and is also expressed on naturally occurring and TGF-*β*-induced Treg cells (n-Treg and i-Treg, resp.) [11–13]. It was recently shown that DR3 expression on B cells was induced by anti-IgM stimulation, although its expression was not detectable on resting B cells [14]. There are several expression differences between human and mouse [9]. DR3 splicing variants of 13 in human [8, 15, 16] and 3 in mice [17] have been identified. Pappu et al. showed that DR3 splicing variants are differentially expressed on T-cell subsets in mice [13].

## 2. Role of TL1A-DR3 Signaling in Cell Fate Determination

TL1A-DR3 signaling induces both NF-*κ*B activation and apoptosis *in vitro* [1, 18, 19]. TL1A-DR3 interaction induces the formation of signaling complexes containing TRADD, TRAF2, and RIP and activates the NF-*κ*B and MAPK pathways (ERK, p38, and JNK). The activation of NF-*κ*B induces c-IAPs, which protect against apoptosis [20]. On the other hand, DR3 overexpression in embryonic cells also induces FADD- and caspase-8-dependent apoptosis [21, 22]. Blocking of TL1A-DR3 signaling by adding NF-*κ*B inhibitors or protein synthesis inhibitors induces apoptosis [20], suggesting that NF-*κ*B activation by TL1A-DR3 interaction is responsible for resistance to apoptosis. Analysis of DR3-deficient mice has shown that DR3 is required for negative selection in the thymus [23]. Inhibition of TL1A-DR3 interaction has shown that TL1A-DR3 signaling is required for effective T-cell immune responses in the target organs of T-cell-mediated autoimmune diseases and inflammatory diseases [24]. Thus, TL1A-DR3 signaling may be involved in lymphocyte homeostasis by modulating either cell death or lymphocyte activation.

## 3. Role of TL1A and DR3 Signaling in the Immune System

### 3.1. Th1

Under TCR stimulation, TL1A induces cell proliferation and the secretion of proinflammatory cytokines (including IFN-*γ*, GM-CSF, and TNF-*α*) in T cells, in particular memory CD4^+^ T cells [1, 25]. Sustained TL1A expression on T cells or dendritic cells leads to an increase in the number of activated CD4^+^ T cells and memory CD4^+^ T cells, whereas sustained TL1A expression on dendritic cells does not stimulate conventional T cells in the absence of TCR stimulation *in vivo,* suggesting that TL1A may act as a costimulator for T cells to regulate inflammatory cytokines and cell proliferation [26, 27]. TL1A synergizes with IL-12/IL-18 to promote IFN-*γ* production in T cells in an antigen-independent manner [25, 28]. TL1A itself cannot directly induce Th1 differentiation of native CD4^+^ T cells, while TL1A-deficient mice show the decrease of IFN-*γ*-producing CD4^+^ T cells [13]. Collectively, it is speculated that TL1A indirectly or synergistically with other cytokines enhances Th1 responses of activated and memory CD4^+^ T cells. The differential T-cell responsiveness for TL1A between naïve and activated/memory T cells might be explained by the regulation of DR3 splicing variants, in particular full-length transmembrane variant, which encodes complete transmembrane DR3 protein. The expression of full-length DR3 mRNA and protein is low level or not detected in naïve and resting T cells [8], whereas it is upregulated in activated T cells [9, 25, 29]. In mice with chronic intestinal inflammation, the transmembrane DR3 expression is upregulated [25].

### 3.2. Th17

Although exogenous TL1A induces IL-2 secretion and responsiveness in T cells [1, 24], IL-2 is a negative regulator for Th17 cells [30]. Therefore, when IL-2 is blocked, exogenous TL1A induces the differentiation of Th17 cells from naïve CD4^+^ T cells stimulated with TCR under Th17 polarization condition *in vitro*. TL1A also induces the proliferation of *in-vitro*-differentiated Th17 effector cells but neither Th1 nor Treg cells even in the absence of TCR stimulation [11, 13]. This differential TL1A responsiveness in T-cell subset might be explained by DR3 expression. DR3 expression is upregulated at a later stage but not early stage of Th17 differentiation. Total DR3 expression is increased in Th17 cells as compared with Th1 and Th2 cells, and full-length transmembrane DR3 expression is increased in Th17 cells as compared with Treg cells [13]. Thus, TL1A-DR3 interaction might preferentially act on Th17 cells and differentially affect the differentiation and maturation of Th17 cells. On the other hand, TL1A inhibits Th17 cell differentiation even in the presence of anti-IL-2 neutralizing antibody *in vitro* [11]. Activation of STAT1 signaling is induced by inflammatory cytokines such as IL-27, IFN-*γ*, and type I IFN and inhibits Th17 differentiation *in vitro* [31]. However, the inhibitory mechanism of Th17 differentiation by TL1A was independent of activation of STAT1 signaling as well as IL-2 signaling [11]. Further, DR3 is dispensable for Th1, Th2, and Th17 differentiation from naïve CD4+ T cells *in vitro* [24]. Thus, the role of TL1A in Th17 differentiation is still controversial *in vitro*. However, TL1A transgenic mice [26, 27], and Th17-mediated autoimmune disease model mice, experimental autoimmune encephalomyelitis [13] and dextran-sulfate-induced chronic colitis [32], show that TL1A-DR3 interaction could positively regulate Th17 cell function *in vivo.* Further research will be required to elucidate the regulatory mechanism of TL1A-DR3 interaction for Th17 cell function *in vitro* and *in vivo*.

### 3.3. Th2

TL1A-DR3 interaction is involved in Th2- as well as Th1- and Th17-mediated immune responses. Transgenic mice that constitutively express TL1A specifically in T cells or dendritic cells develop Th2 cytokine IL-13-dependent small intestinal inflammation [26, 27]. Intranasal immunization with ovalbumin (OVA) together with TL1A in mice induces Th2-mediated immune responses, including OVA-specific IgG1 antibody production in serum, IgA antibody production in mucosal tissues, and the production of Th2 cytokines IL-4 and IL-5 from OVA-restimulated splenocytes *in vitro* [33]. Studies have shown that in DR3-deficient mice, or following blockade of TL1A-DR3 interaction by TL1A neutralization antibodies, OVA-induced lung inflammation is attenuated and Th2 cytokines IL-4, -5, and -13 production is reduced in a mouse model of asthma [10, 24]. In mice with small intestinal inflammation or OVA-induced lung inflammation, NKT cells, activated and memory CD4^+^ T cells, or eosinophils are likely to be a main source of the Th2 cytokines that are induced by the TL1A-DR3 signaling pathway [10, 24, 26, 27], suggesting that TL1A-DR3 signaling in these cells might be a therapeutic target in asthma and ulcerative colitis.

### 3.4. Treg Cells

TL1A transgenic mice show the proliferation and activation of Treg cells in the secondary lymphoid organs and the small intestinal lamina propria [26, 27, 34]. Although exogenous TL1A itself does not affect either n-Treg or i-Treg proliferation *in vitro*, it promotes Treg cell proliferation in the presence of antigen presenting cells with TCR stimulation both *in vitro* and *in vivo* [13, 35], suggesting that TCR signaling is required for costimulation of Treg cells as well as conventional T cells by TL1A. Agonistic anti-DR3 antibodies expand the proliferation of preexisting Treg cells in a manner dependent on TCR and IL-2 signaling *in vivo*, and the expanded Treg cells inhibit OVA-induced lung inflammation [12]. Although Treg cells derived from TL1A-treated mice have highly suppressive activity *ex vivo*, both exogenous TL1A and agonistic anti-DR3 antibodies directly inhibit the suppressive activity of Treg cells *in vitro* [12, 35]. Treg cells derived from mice that constitutively express TL1A under the CD11 promoter attenuate the ability to suppress conventional T cells *in vitro* [26], whereas Treg cells derived from mice constitutively expressing TL1A under the CD2 promoter maintain their suppressive ability [27]. These results suggest that the effect of TL1A-DR3 interaction on T cells might be highly dependent on experimental conditions *in vitro* or the context of the immune response that is being modulated *in vivo*.

### 3.5. NK and NKT Cells

TL1A and agonistic anti-DR3 antibodies synergize with IL-12 and IL-18 to augment IFN-*γ* production in NK cells and NKT cells as well as T cells [28]. The fold-induction of IFN-*γ* production by the addition of TL1A is significantly lower in NK cells and NKT cells than in CD4^+^ T cells and CD8^+^ T cells. The combination of IL-12 and IL-18 drastically increased the DR3 expression in NK cells but minimally in T cells. These data suggest that the augmentation of IL-12/IL-18-induced IFN-*γ* in response to TL1A is differentially induced in T cells and NK cells. TL1A also enhances IL-12/IL-18-induced NK cell cytolytic activity, which is independent of IFN-*γ* production [36], suggesting that TL1A might be an attractive molecule for tumor therapy. Agonistic anti-DR3 antibodies costimulate the proliferation and IL-13 production of NKT cells stimulated with *α*-galactoceramide or anti-CD3 antibodies [10]. NKT-deficient mice, which are resistant to OVA-induced allergic lung inflammation, restore the lung inflammation upon adaptive transfer of wild-type NKT cells, but not after transfer of dominant negative DR3 transgenic NKT cells, suggesting that DR3 signals in NKT cells play an important role for triggering lung inflammation [10].

### 3.6. B Cells

In contrast to T cells, there have been few reports on the significance of TL1A-DR3 interaction in B cells. Membrane-bound TL1A expression on resting B cells was found to be at very low levels in mice [27]. TL1A expression is not induced in B cells either during resting or activated conditions. *In vitro,* TL1A directly reduces B-cell proliferation induced by anti-IgM antibodies and IL-2, whereas it does not affect B-cell proliferation induced by a combination of anti-IgM antibodies and other B-cell-specific stimulators, namely, CpG oligodeoxynucleotide and CD40 ligand [14].

Collectively, these reports indicate that TL1A-DR3 interaction exerts pleiotropic effects on adaptive immune cells, including their activation, proliferation, differentiation, cytokine production, and maintenance.

## 4. Association of *TNFSF15* Gene Polymorphisms with Autoimmune and Inflammatory Diseases

To examine the association of *TNFSF15* gene polymorphisms with autoimmune diseases, Yamazaki et al. performed a genomewide case-control study and found that *TNFSF15* gene polymorphisms are associated with the susceptibility to CD in a Japanese population as well as IBD in a European population [37]. Subsequent replication studies and genomewide association studies have revealed that *TNFSF15* is only one gene that is associated with CD or IBD in both Asian and Caucasian population [38–45]. *TNFSF15* gene polymorphisms are also associated with the severity of CD and IBD in Japanese and Caucasian population, respectively [46–48]. *TNFSF15* haplotypes A and B (which consist of five polymorphisms: rs3810936, rs6478108, rs6478109, rs7848647, and rs7869487) are risk and protective factors, respectively, for susceptibility in both Asian CD and Caucasian IBD patients [37, 39, 49], and haplotype B is a risk factor for severity and antibody status for *E. coli* outer membrane porin C in Jewish CD patients [49, 50]. A polymorphism of *TNFSF15* haplotype A increases promoter activity in stimulated T cells [51], whereas *TNFSF15* haplotype B is associated with increased soluble and membrane TL1A expression in some Jewish CD patients [50]. In addition to IBD, *TNFSF15* gene polymorphism rs4263839 is associated with susceptibility to irritable bowel syndrome and ankylosing spondylitis in Caucasians [52, 53], and we recently found that *TNFSF15* gene polymorphism rs4979462 is associated with susceptibility to PBC in a Japanese population [54]. These findings suggest that *TNFSF15* gene polymorphisms contribute to altered TL1A production, leading to the pathogenesis of autoimmune and inflammatory diseases. In addition to *TNFSF15*, polymorphisms of *IL-23R* and *IL-12A*/*IL-12RB2* are associated with susceptibility to IBD [55, 56] and PBC [57], respectively. TL1A and IL-23 or IL-12 synergistically induce Th1- and Th17-effector cells, implicating the TL1A-IL12/IL-23 pathway in the pathogenesis of both IBD and PBC. Zhang et al. reported that *TNFSF15*, *NOD*, and *IL-23R* are susceptibility genes for leprosy in a Chinese population [58, 59]. These genes are also susceptibility genes in CD, suggesting that CD and leprosy may share a common disease pathway, in particular innate immunity and inflammatory responses.

## 5. Role of TL1A and DR3 in Autoimmune and Inflammatory Diseases

### 5.1. RA

TL1A expression is elevated in the serum, synovial fluid, and synovial tissues of RA patients, in particular patients who are positive for rheumatoid factor (RF). Its expression is correlated with the severity of RA [6, 60]. DR3 gene duplication is more prevalent in RA patients as compared to healthy subjects [61]. Immunohistochemical staining found that TL1A-positive cells in the synovial tissue of RA patients, in particular RF-positive patients, are positive for CD14 and CD68, which are surface markers of macrophages and monocytes [6]. TL1A is induced in human synovial fibroblasts stimulated with TNF-*α* and IL-1*β* [4] and in monocytes stimulated with insoluble immune complexes derived from RA patients [6]. TL1A induces T cells to secrete TNF-*α* and IL-17 under TCR stimulation or Th17 polarization conditions, respectively [4], and it synergizes with IFN-*γ* and augments the production of CXCL8 and matrix metalloproteinase 9 in the human monocytic cell line THP-1 [62]. These inflammatory cytokines and chemokines are associated with RA pathology, and therefore it is possible that TL1A and these inflammatory cytokines form a vicious loop that aggravates RA pathogenesis. Indeed, administration of the anti-TNF-*α* monoclonal antibody adalimumab decreases serum TL1A levels in RA patients [60]. In a mouse model, TL1A administration exacerbated collagen-induced arthritis (CIA) and increased the germinal center formation of spleen and serum anticollagen autoantibodies that are pathogenic in RA [4]. DR3 knockout mice show resistance to development of adverse bone pathology in experimental antigen-induced arthritis (AIA), and TL1A promotes osteoclastogenesis in a DR3-dependent manner in AIA model mice [63], suggesting that DR3 is involved in the generation of osteoclasts at site of bone pathology. Collectively, TL1A-DR3 interaction forms a part of the inflammatory cytokine network and contributes to RA pathogenesis by promoting osteoclastogenesis and the production of inflammatory cytokines and autoantibodies. Furthermore, administration of neutralizing antibodies against TL1A ameliorated both AIA and CIA [63], suggesting that the TL1A-DR3 pathway may be a potential therapeutic target in RA patients.

There has been growing evidence that RA patients are at elevated risk for cardiovascular disease [64]. It was reported that TL1A and its receptors are correlated with atherosclerosis [65]. TL1A regulates the expression of genes implicated in the uptake (scavenger receptors such as SR-A, SR-B1, and CD36) and efflux of cholesterol (ABCG-1, ABCA-1, and ApoE), leading to promotion of foam cell formation in human macrophage [65]. This TL1A-induced macrophage foam cell formation is dependent on DR3. DR3 itself also promotes macrophage foam cell formation by regulating the expression of genes implicated in the uptake and efflux of cholesterol [65]. In addition, it was recently reported that elevated serum TL1A at baseline positively correlates with the progression of atheromatic plaque height in RA patients [66]. A combination of low TL1A and undetectable DcR3 levels in serum at the baseline correlates with decrease of new atheromatic plaques in carotid arteries and/or femoral arteries of RA patients over a follow-up period of 3.5 years, suggesting that serum TL1A and DcR3 levels might predict a preserved atherosclerosis profile in carotid and/or femoral arteries. The expression of DR3 mRNA but neither TL1A nor DcR3 shows a trend to evaluation in atheromatous plaques of arterial tissue. Collectively, these data highlights that TL1A-DR3/DcR3 signaling is involved in chronic inflammation and atherosclerosis.

### 5.2. Human IBD

In IBD patients, TL1A expression is increased in both serum and intestinal tissues and is correlated with the disease activity [67–69]. DR3 expression is also increased in lymphocytes, in particular T cells, in the intestinal lamina propria in these patients [68]. Interestingly, TL1A-expressing cells in the lamina propria are macrophages and CD4^+^ or CD8^+^ T cells in CD patients, whereas they are mainly plasma cells in UC patients [68]. The diversity of TL1A expression might reflect differences in the pathogenesis of CD and UC. TL1A expression is increased only in lamina propria CD14^+^ macrophages (but not peripheral monocytes or monocyte-derived macrophages) in CD patients but not in UC patients or healthy subjects. The membrane-bound expression of TL1A is induced in lamina propria CD14^+^ macrophages stimulated by commensal bacteria such as *E. coli* [70]. In UC, IgG-producing plasma cells were found to infiltrate areas of mucosal inflammation, and IgG immune complex stimulation increased TL1A expression in macrophages in the lamina propria [71]. These findings indicate that mononuclear phagocytes are likely to be a major source of TL1A in inflamed loci in the intestines of IBD patients. In CD patients, exogenous TL1A and agonistic anti-DR3 antibodies augment IFN-*γ* production in lamina propria mononuclear cells as well as peripheral blood mononuclear cells [68, 69]. TL1A synergizes with IL-12/IL-18 and induces IFN-*γ* production in lamina propria CCR9^+^ T cells derived from CD patients, which is thought to be implicated in the pathogenesis of CD [72]. Exogenous TL1A and IL-23 synergistically induce IFN-*γ* and IL-17 secretion in lamina propria CD4^+^ T cells. TL1A enhances the differentiation of IL-17- and IFN-*γ*/IL-17-producing Th17 cells when naïve CD4^+^ T cells are stimulated with lamina propria macrophages derived from CD patients [70]. These reports demonstrate that TL1A-DR3 interaction may contribute to Th1- and Th17-mediated responses that are characteristic of CD.

### 5.3. IBD Mouse Model

TL1A is increased in the intestines of IBD model mice, including dextran-sodium-sulfate (DSS-) induced chronic colitis mice and TNF^ΔARE^ chronic ileitis mice. Its major source is likely to be dendritic cells in mesenteric lymph nodes and small intestinal lamina propria mononuclear cells [25, 32]. Mice with constitutive TL1A expression in antigen-presenting cells and T cells show intestinal inflammation and colonic fibrosis with a high percentage of T cells that are positive for CCR9 and CCR10, both of which are gut-homing chemokines in T cells [34]. As with TL1A, DR3 expression is increased in DSS-induced chronic colitis mice, and its transmembrane splicing variant is increased in correlation with inflammation in chronic ileitis mice, TNF^ΔARE^ mice, and SAMP1/Yit Fc mice [25, 32]. Administration of blocking anti-TL1A monoclonal antibodies inhibits DSS-induced colonic inflammation in mice [32], and DR3-deficient mice are protected from intestinal inflammation even after colitis induction [27]. In mice with 2,4,6-trinitrobenzenesulfonic acid- (TNBS-) induced colitis, colonic inflammation is inhibited by administration of anti-TL1A or anti-DR3 antibodies [27]. These reports demonstrate that TL1A-DR3 interaction plays an important role in the pathogenesis of these chronic intestinal inflammatory conditions in mice. TL1A synergizes with IL-12 or IL-23 and induces both IFN-*γ* and IL-17 secretion in CD4^+^ T cells derived from gut-associated lymphoid tissue (GALT) of DSS-induced chronic colitis mice [32]. IL-23 itself or IL-23 in combination with TL1A induces IFN-*γ*/IL-17 double-positive CD4^+^ T cells that are known to be colitogenic [32]. Sustained TL1A expression in T cells or dendritic cells promotes goblet cell and Paneth cell hyperplasia in the small intestine in mice [26, 27, 34]. Hyperplasia of goblet cells and Paneth cells is associated with elevated Th2 cytokine production [73, 74]. In mice with sustained TL1A expression, IL-13 and IL-17 expression is increased in mesenteric lymph node cells, ileum, and CD4^+^ T cells isolated from the lamina propria. Administration of antagonistic anti-IL13 antibodies but not anti-IL17 antibodies attenuates intestinal inflammation in mice constitutively expressing TL1A [27], suggesting that IL-13 plays an important role in the small intestinal inflammation induced by sustained TL1A expression on T cells or dendritic cells. Although agonistic anti-DR3 antibodies and glycosphingolipid enhance the production of Th2 cytokines IL-13 and IL-4 in NKT cells [10], mice with sustained TL1A expression and small intestinal inflammation show decreased NKT cells and increased activated and memory CD4^+^ T cells, suggesting that in these mice the major source of IL-13 is likely to be activated and memory T cells [26, 27]. In human, CD is associated with the Th1/Th17 cytokines IL-12 and IL-23, and UC with the Th2 cytokine IL-13 [75]. Taken together, these findings in IBD model mice provide evidence that TL1A-DR3 interaction may contribute to both Th1/Th17 and Th2 signaling pathways in human IBD.

### 5.4. Psoriasis

Bamias et al. reported an association of TL1A and its two receptors with psoriasis [76]. TL1A is mainly expressed in keratinocyte, basal cells, vascular cells, and infiltrating inflammatory cells of psoriatic skin but is rarely expressed in those of normal skin. DR3 and DcR3 are expressed in normal skin and are upregulated in psoriatic skin. The expression levels of TL1A and its two receptors are upregulated in lesional skin as compared to nonlesional skin in psoriasis patients, suggesting that TL1A and its two receptors may be involved in the pathogenesis of psoriasis. Immunohistochemical staining shows that TL1A localizes at nuclear region in inflammatory and fibroblast-like cells in psoriasis patients, although previous studies reported that TL1A localizes at cytoplasmic region in inflamed tissues of several autoimmune diseases [6, 68]. In this paper, the unique nuclear location is also confirmed in synoviocytes and inflammatory cells in RA patients. Further study is needed to show the significance of nuclear TL1A localization. TL1A transgenic mice develop chronic intestinal inflammation and a minority of the mice also develop ulcerative skin lesions and arthritis. Concurrent rate among IBD, RA, and psoriasis is high in human. These reports might provide the evidence that TL1A is one of common denominators of gut, joint, and skin inflammation.

### 5.5. PBC

We recently found that TL1A expression is increased in both serum and liver tissues of PBC patients. In the liver, TL1A expression is positive for infiltrating mononuclear cells, endothelial cells, Kupffer cells, and biliary epithelial cells. Serum TL1A levels are decreased in early-stage but not late-stage patients being treated with ursodeoxycholic acid, the only therapeutic drug for PBC approved by the Food and Drug Administration, suggesting that TL1A has a potential to be a new serum marker and therapeutic target for PBC [77].

## 6. The Association of DcR3 with Autoimmune and Inflammatory Diseases

DcR3, also known as TR6, and M68, is a member of the TNFRSF. DcR3 consists of 300 amino acids lacking a transmembrane domain of TNFRSF and be released as secreted protein. DcR3 functions as a decoy receptor for TL1A as well as FasL and LIGHT and inhibits these ligands mediated apoptosis and lymphokine secretion [78–81]. DcR3 is induced in human antigen-presenting cells such as monocytes and myeloid dendritic cells and intestinal epithelial cells lines by lipopolysaccharide or lipoteichoic acid and is also induced in human dermal microvascular endothelial cells by TNF-*α* and IL-1*β* [82, 83]. DcR3 modulates the differentiation and maturation of monocyte, macrophage, and dendritic cells, polarization of naïve T cells into Th-2 immune response [84], and the negative regulation for activation of B cells by TLR ligands [85]. DcR3 is rarely detectable in serum of healthy subjects, whereas its expression is increased in that of various autoimmune and inflammatory diseases such as IBD [67, 86], SLE [78], RA [60], PBC [77], silicosis [87], viral infections [82], renal failure [88], and atopic dermatitis [89] as well as cancer [90]. DcR3 is not found in mouse genome, suggesting that additional complexity of TL1A-DR3 pathway in human as compared to mouse. DcR3 protects the development of autoimmune diabetes [91, 92], IgA nephropathy [93], and crescent glomerulonephritis [94] model mice, while DcR3-transgenic mice develop SLE-like syndrome [95]. These reports suggest that DcR3 also plays an important role in the pathogenesis of autoimmune and inflammatory diseases.

## 7. Conclusion

**Figure 1:**
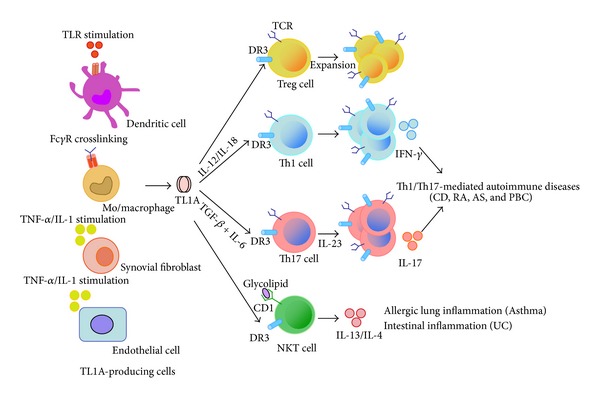
TL1A connects innate immune responses to adaptive immune responses and is critically involved in the induction of autoimmune and inflammatory diseases.

TL1A expression is transiently induced by inflammatory cytokines, TLR ligands, enteric bacteria, and Fc*γ*R crosslinking in antigen-presenting cells or non-immune cells. Although TL1A was initially characterized as a costimulator for inducing cell proliferation and cytokine secretion in T cells, there is growing evidence that TL1A has pleiotropic effects such as cell death, differentiation, and maintenance of lymphocytes, as well as osteoclastogenesis and atherosclerosis. Increased TL1A expression and/or TL1A (*TNFSF15*) gene polymorphisms are associated with the pathogenesis of various autoimmune and inflammatory diseases. Analysis of murine models of autoimmune diseases and TL1A or DR3 transgenic mice suggests that TL1A-DR3 interaction plays an important role in local inflammation of T-cell-dependent autoimmune diseases. Thus, TL1A connects innate immune responses to adaptive immune responses and is critically involved in the induction of autoimmune and inflammatory diseases (Figure 1), suggesting that inhibition of TL1A-DR3 interaction could be an effective therapeutic strategy for ameliorating local inflammation in target organs of individuals with autoimmune diseases.
